# An anthropogenic habitat within a suboptimal colonized ecosystem provides improved conditions for a range‐shifting species

**DOI:** 10.1002/ece3.3739

**Published:** 2018-01-01

**Authors:** Zachary J. Cannizzo, Sara R. Dixon, Blaine D. Griffen

**Affiliations:** ^1^ Marine Science Program School of the Earth, Ocean, and Environment University of South Carolina Columbia SC USA; ^2^ Department of Biology Brigham Young University Provo UT USA

**Keywords:** *Aratus pisonii*, climate change, habitat analog, mangrove, salt marsh, thermal habitat

## Abstract

Many species are shifting their ranges in response to the changing climate. In cases where such shifts lead to the colonization of a new ecosystem, it is critical to establish how the shifting species itself is impacted by novel environmental and biological interactions. Anthropogenic habitats that are analogous to the historic habitat of a shifting species may play a crucial role in the ability of that species to expand or persist in suboptimal colonized ecosystems. We tested if the anthropogenic habitat of docks, a likely mangrove analog, provides improved conditions for the range‐shifting mangrove tree crab *Aratus pisonii* within the colonized suboptimal salt marsh ecosystem. To test if docks provided an improved habitat, we compared the impact of the salt marsh and dock habitats on ecological and life history traits that influence the ability of this species to persist and expand into the salt marsh and compared these back to baselines in the historic mangrove ecosystem. Specifically, we examined behavior, physiology, foraging, and the thermal conditions of *A. pisonii* in each habitat. We found that docks provide a more favorable thermal and foraging habitat than the surrounding salt marsh, while their ability to provide conditions which improved behavior and physiology was mixed. Our study shows that anthropogenic habitats can act as analogs to historic ecosystems and enhance the habitat quality for range‐shifting species in colonized suboptimal ecosystems. If the patterns that we document are general across systems, then anthropogenic habitats may play an important facilitative role in the range shifts of species with continued climate change.

## 1 INTRODUCTION

Climate change is forcing or encouraging many species to shift their geographic ranges (Canning‐Clode, Fowler, Byers, Carlton, & Ruiz, 2011; Sorte, Williams, & Carlton, 2010; Walther et al., 2002). These shifts are often associated with the simultaneous shifts of ecosystem foundation species (Walther, 2010). However, differential shifting rates between the ecosystem foundation species and other species in the community can occur and may have cascading effects on community structure and ecosystem function. When such a mismatch in shifting rates occurs, it can result in a species colonizing a new ecosystem which it has never previously inhabited (Schweiger, Settle, & Kudrna, 2008). Colonization of new ecosystems as a result of different shifting rates is expected to increase as climate change continues (Schweiger et al., 2008; Walther, 2010).

While there has been abundant discussion on the importance of corridors in aiding range‐shifting species through increasing habitat connectivity (Hannah, 2001; Heller & Zavaleta, 2009; Krosby, Tewksbury, Haddad, & Hoekstra, 2010; Williams, Shoo, Isaac, Hoffmann, & Langham, 2008), little work has been done to determine how these shifts impact the species themselves. This is particularly true of range shifts which result in the colonization of new ecosystems. A range shift into an ecosystem that a species has not previously inhabited exposes the colonizing species to novel biological and environmental interactions. Due to the complexity of these interactions, predicting how they will impact both the colonized ecosystem and the colonizing species can be difficult. The invasion literature contains abundant research on the impact of novel species on colonized ecosystems (Mooney & Cleland, 2001; Salo, Korpimäki, Banks, Nordström, & Dickman, 2007; Vilá et al., 2011 and references therein). Yet, the impact of novel habitats on colonizing species is relatively understudied (but see Phillips, Brown, & Shine, 2010), likely because most studies of novel species–ecosystem interactions are found in the invasion literature where the invader is viewed as unnatural and therefore undesirable.

Among other factors, a colonizing species may find itself in an ecosystem that differs greatly from its historic ecosystem in foundation species, structure, food sources, and environmental stressors. Barring preadaptation (Hamilton, Okada, Korves, & Schmitt, 2015), these differences are likely to result in suboptimal conditions for the colonizing species (Holt, Barfield, & Gomulkiewicz, 2005; Keller & Taylor, 2008). In fact, novel biotic and abiotic interactions result in the failure of the majority of introduced species to establish populations (Williamson, 1996; Zenni & Nuñez, 2013 and references therein). While those colonizing species that can establish a foothold may be able to adapt to these novel interactions over time (Hamilton et al., 2015; Kaweki, 2008; Knope & Scales, 2013), early generations will likely display symptoms of living in suboptimal conditions that will affect their fitness and potentially limit their further expansion into the new ecosystem.

Despite the difficulties faced by a colonizing species, pockets of habitat which replicate some of the conditions of its historic ecosystem may exist within the colonized ecosystem. These pockets of habitat can be thought of as analogs to the historic ecosystem of the colonizing species. Thus, we adopt the terms “habitat analog” and “analogous habitat” from the urban and reconciliation ecology literature (sensu Lundholm & Richardson, 2010). Habitat analogs have received some attention as artificial habitats found in highly altered ecosystems that replicate conditions experienced by species in their native ecosystems (Lundholm & Richardson, 2010 and references therein). These habitats range from quarries (Tropek & Konvička, 2008; Tropek et al., 2010) to urban rubble (Grant, 2006) and often provide habitat and refuge for species that could not otherwise thrive in the surrounding ecosystem (Chester & Robson, 2013; Lundholm & Richardson, 2010). While the terms habitat analog and analogous habitat have predominantly been used to refer to those habitats found within highly altered ecosystems, the terminology is directly applicable to patches of habitat within natural, but suboptimal, colonized ecosystems that more closely resemble the historic ecosystem of the colonizer. Whether natural or anthropogenic, analogous habitats and other refuges may provide benefits such as a more favorable thermal environment (Mosedale, Abernethy, Smart, Wilson, & Maclean, 2016; Wilson et al., 2015), predation refuge (Dumont, Harris, & Gaymer, 2011), and higher quality foraging. Any of these benefits could help a species persist or expand more rapidly into an otherwise suboptimal ecosystem. Thus, these habitat analogs have the potential to play a crucial role in current and future range shifts. However, the impact of analogous habitats and other refuges on range‐shifting species within colonized ecosystems is relatively understudied (but see Wilson et al., 2015).

The mangrove tree crab *Aratus pisonii* offers an ideal opportunity to examine the impacts of both a colonized ecosystem and a potential analogous habitat on a range‐shifting species. This arboreal crab is historically associated with Neotropical mangrove forests dominated by the red mangrove *Rhizophora mangle* (Wilson, 1989). However, its climate‐mediated northward range expansion has recently outpaced that of the mangrove ecosystem resulting in the colonization of salt marshes in the southeastern United States (Riley, Johnston, Feller, & Griffen, 2014). The salt marsh, which is dominated by the grass *Spartina alterniflora*, differs greatly from the mangrove forests where *A. pisonii* has historically been found. The mangrove provides a shaded habitat with tall vertical structure and easy access to the primary food source of *A. pisonii*,* R. mangle* leaves (Beever, Simberloff, & King, 1979; López & Conde, 2013), which are absent in the salt marsh. Thus, *A. pisonii* in the salt marsh find themselves in an ecosystem which differs greatly in structure and foraging opportunities from that to which they are adapted. As a result, *A. pisonii* in the salt marsh display smaller body sizes, smaller clutch sizes, and lower larval quality than conspecifics in the mangrove (Riley & Griffen, 2017). Thus, it appears that compared to the historic mangrove, the salt marsh is a suboptimal habitat for *A. pisonii*. However, *A. pisonii* is also found on the anthropogenic habitat of docks within the salt marsh.

Analogous habitats confer benefits on a species by being in some way similar to its historic ecosystem. Docks may fit this criterion within the salt marsh as they provide *A. pisonii* with a shaded habitat and vertical structure more similar to the historic mangrove as well as easy access to food in the form of abundant fouling communities. While mangrove leaves are not available in the dock habitat, animal material, which is abundant on docks in the form of fouling communities, is a high‐quality food source (Riley, Vogel, & Griffen, 2014) that is preferred by *A. pisonii* over mangrove leaves (Erickson, Feller, Paul, Kwiatkowski, & Lee, 2008). Easy access to a high‐quality food source could be a boon to *A. pisonii* as the quantity and quality of diet play crucial roles in the energetics and life history of an individual (Charron et al., 2015; Wen, Chen, Ku, & Zhou, 2006). The shaded habitat provided by the dock itself, which is similar to the shade provided by a mangrove canopy, may be an additional benefit as the thermal habitat experienced by an organism has a direct impact on its physiology and life history (Huey, 1991; Leffler, 1972), especially when warmer than optimal (Gillooly, Brown, West, Savage, & Charnov, 2001). Thus, the structure, food, and shade provided by docks may allow them to provide improved habitat for *A. pisonii* within the suboptimal salt marsh. The use of anthropogenic structures to provide favorable habitat for species experiencing adverse effects of climate change has been proposed (Shoo et al., 2011) and implemented (Mitchell, Kearney, Nelson, & Porter, 2008) as an aspect of adaptive management (Heller & Zavaleta, 2009). However, these structures have always been designed to counteract negative impacts experienced by species in either their historic or highly degraded ecosystems. Unlike the use of shade‐cloth shelters (Mitchell et al., 2008) and artificial burrows (Souter, Bull, & Hutchinson, 2004), docks represent an anthropogenic habitat found in a colonized natural ecosystem that was not intended to improve habitat conditions.

We examine the impact of the salt marsh and dock habitats on ecological and life history traits of *A. pisonii* that influence both individual performance and the ability of this species to persist and expand into the salt marsh. This includes aspects of behavior related to diet and energy storage, thermal conditions experienced by *A. pisonii*, and an exploration of dietary intake and quality in each habitat. We compare individuals from the colonized habitats (salt marsh and dock) to each other and to a baseline of conspecifics from the historic mangrove ecosystem. We test the overarching hypothesis that, in each aspect, *A. pisonii* found on docks within the salt marsh will be more similar to conspecifics in the historic mangrove than to those in the surrounding salt marsh.

## 2 METHODS

### 2.1 Study species

*Aratus pisonii* is a mangrove‐associated crab found throughout the Neotropics (Rathbun, 1918; Warner, 1967). This largely arboreal semiterrestrial crab has an ecology that is closely tied to the mangrove trees themselves (Beever et al., 1979; Warner, 1967). In fact, while it will feed opportunistically on high‐quality animal material (Beever et al., 1979; Erickson et al., 2008), its primary food source is fresh mangrove leaves, specifically from the red mangrove *R. mangle* (Beever et al., 1979; López & Conde, 2013). Individuals maintain strong site fidelity to individual trees, a behavior lost in the salt marsh, from which they tend to move only a short distance (Cannizzo & Griffen, 2016). Despite this fidelity, this crab is not aggressively territorial, it is not uncommon to see numerous individuals in close proximity, and the species maintains a size and sex‐based social hierarchy largely through ritualistic displays (Warner, 1970). Further, this species is largely terrestrial, returning to the water only to wet its gills and release larvae, and even exhibits a characteristic climbing behavior to avoid aquatic predators when the tide rises (Warner, 1967; Wilson, 1989).

### 2.2 Site description

**Figure 1:**
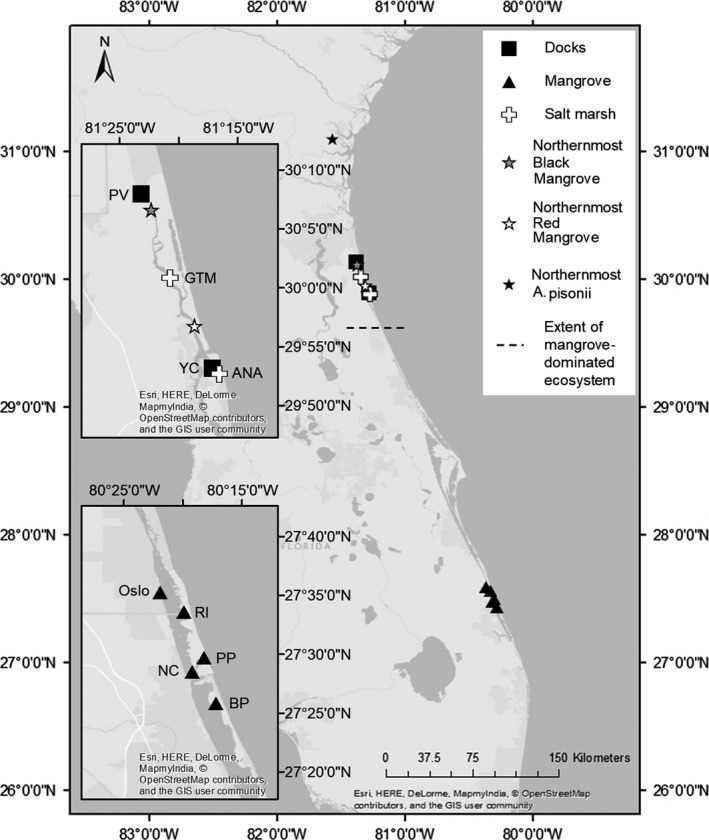
Map of the location of study sites, northernmost *Aratus pisonii* (Riley, Johnston et al., 2014), and northernmost black (*Avicennia germinans*) and red (*Rhizophora mangle*) mangroves (Williams, Eastman et al., 2014; Williams, Lundholm et al., 2014). The map also displays a point delineated as the extent of the mangrove‐dominated ecosystem. While the transition from mangrove to salt marsh exists as a mosaic‐like ecotone, this location represents an area with roughly 50:50 mangrove:salt marsh coverage (Rodriguez, Feller, & Cavanaugh, 2016; IC Feller pers. com.). North of this line, mangroves can still be found but are progressively more isolated and exist as individuals or small patches within a salt marsh‐dominated ecosystem

We examined *A. pisonii* in mangrove forests in the vicinity of Fort Pierce, Florida, while individuals in the salt marsh and dock habitats were found in the vicinity of Saint Augustine, Florida (Figure 1; Table S1). The mangrove sites are within the historic range of *A. pisonii* (Rathbun, 1918; Warner, 1967), while salt marsh and dock sites represent habitats within the recently colonized region (Riley, Johnston et al., 2014). The sites chosen were selected as they are representative of their respective habitat type. Studied salt marsh sites were always at least 0.75 km from the nearest dock to prevent the possibility of examining crabs that have access to the dock habitat. While two salt marsh sites and one dock site were south of the northernmost mangrove (Figure 1), mangroves are scarce in this salt marsh‐dominated ecosystem and tend to exist only in small isolated pockets of individuals. Further, only one site of each habitat is south of the northernmost red mangrove, the species to which the ecology of *A. pisonii* is most closely tied in the mangrove ecosystem (Beever et al., 1979; Warner, 1967). While it was impossible to ensure that there was no movement between the dock and salt marsh for crabs examined on docks, crabs tend to exhibit little movement from a central foraging area (Cannizzo & Griffen, 2016). Further, even if there is some movement between the habitats, this would result in a conservative test of our hypotheses by minimizing observed differences.

### 2.3 Behavioral observations

We observed the behavior of individual crabs *in situ*. In each habitat, we collected groups of five adult *A. pisonii* by hand and determined the sex and carapace width (to the nearest 0.1 mm) of each individual. The groups of crabs were made up of the first five individuals that we encountered and could capture and were drawn from all accessible habitats. We then painted the carapace of each crab an identifying color with nail polish to aid in identification and visibility. Preliminary experiments determined that painting the carapaces of crabs did not alter their behavior or thermal properties. Following a short period of observation to ensure normal behavior, we released the crabs onto a single tree within 10 m of the collection tree of all individuals (mangrove), onto separate *S. alterniflora* stalks within 10 m of the area of collection (salt marsh), or onto the same piling (dock) of the dock where all individuals were captured. Releasing crabs near their capture location allowed for observation while also ensuring as near a natural distribution of crabs as possible. To avoid immediate retreat into holes, release in the salt marsh occurred during the rising tide when the crabs had no access to the sediment.

In all habitats, *A. pisonii* climbs structure as the tide rises to remain out of the water and will even leave occupied shelter to do so (personal observation). Thus, we observed crabs in the mangrove and salt marsh habitats from the time they lost access to the sediment until the receding tide once again allowed access to the sediment (~6 hr depending on site and day). In contrast, in the dock habitat, crabs generally lack access to the sediment throughout the tidal cycle. To obtain an observational period similar to that of the other habitats, we therefore observed crabs on docks from 3 hr before slack high tide until 3 hr after slack high tide. We watched crabs from a distance using binoculars to avoid impacting their behavior and monitored the individuals continuously throughout the observational period. The observational location was chosen to maximize visibility, and the observer was free to move if increased visibility was necessary. Behavior was recorded every 5 min and at every change in behavior within those 5‐min intervals as one of four categories: feeding, sitting, moving, or not‐visible (Table 1). Each group of five crabs was only observed for behavior once, and only one group of crabs was observed on any given day. All observations occurred from May through August.

**Table 1:** Ethogram describing the behavioral categories assigned while observing *Aratus pisonii*

Behavior	Description
Feeding	The crab is observed actively moving its claws from a food item or substrate to its mouth.
Moving	The crab is actively moving along a substrate and not feeding. Other energy‐expending nonfeeding activities, such as ritual aggression, were also classified under moving as they represent an expenditure of energy. However, these activities were rare and short‐lived
Sitting	The crab is not actively moving, feeding, or participating in any activity
Not‐visible	The crab is not visible to the observer

We separated the observations into ebb and flood tidal periods to examine differences in foraging behavior as crabs gained or lost access to food sources on the sediment and wet habitat structure. To avoid biasing the data with crabs that were not visible for long periods, we also removed data from individuals that were not visible for more than 66% of the tidal period. This correction resulted in the observation of 38, 55, and 39 individuals during flood tide and 41, 54, and 39 individuals during ebb tide in the mangrove, salt marsh, and dock habitats, respectively. Unless otherwise stated, these individual crabs were treated as the replicates for all associated statistical analyses.

To test for the effects of multiple biological and environmental variables on the proportion of time spent feeding during flood or ebb tide, we ran a generalized linear mixed model with a binomial error distribution. We included carapace width, sex, habitat, air temperature, and tide (ebb or flood) as explanatory variables. We also included the individual crab ID as a random factor to account for the multiple observations of individual crabs (ebb and flood tide) and weighted the model by the total time of observation for each individual. Additionally, we explored the proportion of time *A. pisonii* spent moving by employing a similar generalized linear mixed model but with the proportion of time individuals spent moving as the response variable.

### 2.4 Exposure to thermal microhabitats

To explore the thermal conditions experienced by *A. pisonii* in each habitat, we compared the solar exposure they experienced. We did this by recording the position of crabs as in sun or shade during the behavioral observations described above and calculating the proportion of time they spent in the sun. To confirm the inherent assumption that individuals experience higher temperatures while in the sun, we placed HOBO thermal data loggers underneath a dock, and in a nearby salt marsh at the same site attached to a wooden dowel high enough to remain out of the water. These loggers simultaneously gathered temperature data every minute from noon on 8 September 2016 to noon on 11 September 2016. The logger data were not collected coincident with observations of crabs as it was not intended to measure the exact temperatures crabs experienced but relative differences between temperatures in the sun and shade. While we took advantage of the structural differences between these habitats to obtain data pertaining to temperature exposure while crabs are in the sun (salt marsh logger) and shade (dock logger), these measures do not necessarily represent the thermal conditions experienced by all crabs in each of the two habitats at all times. Rather, as the dock and mangrove provide shaded canopies and the salt marsh does not, they represent the difference in the thermal conditions most often experienced by the crabs in each habitat.

To further examine the thermal habitat experienced by the observed crabs, we used a FLIR instruments C2 compact thermal imaging camera to take a thermal image of each visible marked crab every 15 min throughout the observational period. The days when crabs were observed took place over a wider range of air temperatures, which was measured on site, in the mangrove and salt marsh habitats than on docks. Thus, to avoid the confounding factor of relatively cooler air temperatures in these habitats, only thermal pictures taken on days which had an average air temperature >29°C were examined. This temperature represented the lower bound of air temperatures on days crabs were observed in the dock habitat. Along with the elimination of photographs where no crabs were visible, this resulted in the analysis of 455, 294, and 289 thermal photographs from the salt marsh, mangrove, and dock habitats, respectively. We then employed the program FLIR tools to obtain the temperature at the center of the carapace of each crab.

We suspected that the proportion of time crabs spent in both the water and the sun would impact their body temperature, so we calculated these values for all individuals for which we had thermal photographs. We compared these values between habitats using an ANOVA followed by a Tukey's HSD test for multiple comparisons. Unless otherwise stated, we implemented this statistical method for all subsequent comparisons made between and within habitats.

To explore the factors that influence crab temperature, we averaged the recorded body temperature of individual crabs over the course of an observational period. We expected that the solar radiation experienced by crabs over the course of an observational period (~6 hr depending on site and day) would impact their body temperature. Thus, to examine the impact of solar exposure on crab temperature, we obtained short‐ and long‐wave solar radiation from the NCEP North American Regional Reanalysis (NARR). NARR has a resolution of 32 km and calculates solar radiation in 3‐hr intervals. We obtained the solar radiation at the grid point closest to each site and averaged the sum of the short‐ and long‐wave solar radiation over the observational period. This number, in W/m^2^, was then multiplied by the number of seconds the crab was observed to spend in the sun to obtain a relative measure of the solar energy experienced over the observational period. This calculated variable will hereafter be referred to as “solar exposure”. We then ran a mixed effects linear model with habitat, proportion of time in water, solar exposure, and ambient air temperature as explanatory factors for the averaged crab body temperatures, which were included as the response variable in the model. We also ran a similar model with the average difference between crab body temperature and the ambient air temperature as the response variable. This model allowed us to analyze the ability of crabs in each habitat to maintain a body temperature cooler than ambient and explore the factors that impact this ability. In both models, the continuous explanatory variables were *z*‐scored to facilitate comparison of their relative impacts on the response variable. Due to the site‐fidelity behavior of *A. pisonii* (Cannizzo & Griffen, 2016), some crabs were photographed on multiple days. Thus, to account for these multiple observations, crab ID was included in the models as a random factor. These models allowed us to explore the impact of these factors on both crab body temperatures and cooling on the timescale on which the explanatory factors were available and meaningful. Finally, we ran linear regressions to determine whether there were relationships between the proportion of time individuals spent in the water and sun as well as the time spent in water and solar exposure.

### 2.5 Diet and energy storage

To examine diet indices and the investment of *A. pisonii* into energy storage, we collected individuals from each habitat during the summers of 2015 and 2016. On each of nine randomly selected days in each habitat, 15 individual adult *A. pisonii* were collected by hand and immediately placed on dry ice. In the mangrove and salt marsh, we collected these crabs in three groups of five at three distinct tidal periods: just after losing access to the sediment on the flood tide, at slack high tide, and just before regaining access to the sediment on the ebb tide. This resulted in collection times ~3 hr apart. Due to the constant lack of access to sediment in the dock habitat, we collected crabs 3 hr before, at, and 3 hr after slack high tide. As in the behavioral observations, the first five crabs we encountered were collected at each of these tidal periods. This collection regime resulted in a total of 135 crabs from each habitat (45 from each tidal period) which were kept frozen until dissection. No measured indices differed between years, and thus, data were pooled across years for analysis.

Based on preliminary observations in the laboratory, the gut clearance time of *A. pisonii* is ~3 hr. Therefore, our collection regime allowed for the analysis of diet when crabs had access to the sediment (collected on the flood tide), when crabs only had access to unsubmerged habitat (collected at slack high tide), and when crabs had access to recently submerged habitat (collected on the ebb tide). Prior to dissection, we determined the sex and carapace width (to the nearest 0.1 mm) of each crab.

We ascertained the gut fullness of each crab to obtain a snapshot of the quantity of food consumed during each tidal period by removing the gut contents and drying them at 60–70°C to constant weight. We standardized gut fullness by dividing the mass of the gut contents by the volume of the gut ($V=a\left(\sqrt{2}/12\right)\times \text{Gut}\;{\text{width}}^{3}$, where *a* is a correction factor of 0.92 for crabs [Griffen & Mosblack, 2011]). We then employed a two‐way ANOVA to compare the standardized gut fullness between tidal periods within and between habitats. Due to inclement weather during one observation day in the dock habitat, crabs were collected without regard for tidal period. This lead to only 120 crabs from the dock, 40 per tidal period, being analyzed for gut fullness. As this was the only dissection parameter dependent on time of collection (see below), only gut fullness was impacted by this reduced sample size.

In addition to diet quantity, we explored long‐term diet quality by measuring the cardiac stomach of each crab to the nearest 0.1 mm and comparing the gut‐width:carapace‐width ratio between habitats. In crabs, this ratio is a proxy for long‐term diet quality with a smaller ratio corresponding to a higher quality diet that likely contains more animal material (Griffen & Mosblack, 2011).

To examine the proportional energetic investment into energy storage by conspecifics in each habitat, we separated and dried the primary energy storage organ (hepatopancreas) (Parvathy, 1971) and the somatic tissue of each crab. To compare energetic investment between habitats, we calculated the hepatosomatic index (HSI) of each crab as the ratio of the dry weights of the hepatopancreas and the somatic tissue, which is a common measure of energy stores in crustaceans (Griffen, Vogel, Goulding, & Hartman, 2015; Kennish, 1997; Riley, Vogel et al., 2014; Sánchez‐Paz, García‐Carreño, Hernández‐López, Muhlia‐Almazán, & Yepiz‐Plascencia, 2007). However, HSI is dependent on both sex and reproductive stage (e.g., a female will have a lower HSI when carrying eggs; Belgrad, Karan, & Griffen, 2017). Thus, we grouped crabs as male, gravid female, or nongravid female and compared the HSI of these groups between habitats. Due to a problem in transportation, the legs of crabs from two tidal periods on one day from the mangrove became detached and mixed. This made it impossible to reliably obtain a weight for somatic tissue from these 10 crabs resulting in a reduced sample size of 125 crabs from the mangrove analyzed for HSI. As this was the only parameter that incorporated somatic weight, it did not affect the sample size of any other analysis.

### 2.6 Statement of animal rights

All applicable institutional and/or national guidelines for the care and use of animals were followed.

## 3 RESULTS

### 3.1 Demographics

*Aratus pisonii* in the salt marsh habitat were smaller (CW ± *SD* = 12.97 ± 1.57 mm) than conspecifics in the mangrove (17.95 ± 3.12 mm) and dock (17.83 ± 2.09 mm) habitats (ANOVA, *F*_2_ = 314.9, *p* < .001; Tukey's HSD, *p* < .001, Figure S1). However, individuals found in the dock habitat did not differ in size from conspecifics in the mangrove (Tukey's HSD, *p* = .850, Figure S1).

### 3.2 Behavioral observations

**Figure 2:**
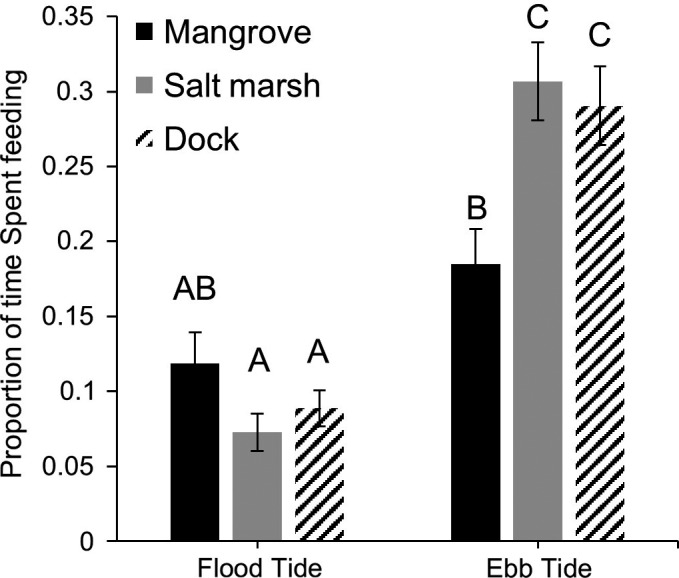
The proportion of time spent feeding ± SE by *Aratus pisonii* in the mangrove, salt marsh, and dock habitats before and after slack high tide. Groups that are significantly different are denoted by different letters

For the results presented below, “estim.” refers to the parameter estimate for the statistical model being reported. The proportion of time *A. pisonii* spent feeding was lower in the mangrove (Prop. time ± *SD* = 0.152 ± 0.139) than the dock (0.190 ± 0.162; GLM, estim. = −0.754, *z* = −3.01, *p* = .003) and salt marsh habitats (0.189 ± 0.190; GLM, estim. = 0.792, *z* = 3.28, *p* = .006) but did not differ between the dock and salt marsh (GLM, estim. = −0.218, *z* = −0.94, *p* = .349). Time spent feeding was not affected by carapace width or sex (GLM, estim. = −0.042, *z* = −0.99, *p* = .326; estim. = 0.382, *z* = 1.87, *p* = .062, respectively), but was influenced by a number of environmental factors. Feeding decreased as air temperature increased (GLM, estim. = −0.135, *z* = −6.90, *p* < .001), but increased as the tide fell and foraging on recently submerged structure became possible (GLM, estim. = 1.460, *z* = 43.28, *p* < .001). Time spent feeding also differed within habitats and was contingent on the tidal period (two‐way ANOVA, Habitat × Tide, *F*_2_ = 8.664, *p* < .001; Tukey's HSD, *p* < .05, Figure 2). Additionally, foraging depended on interactions between the tide and habitat. After slack tide, crabs in the salt marsh exhibited a 1.4‐fold greater increase in feeding than crabs on docks (GLM, estim. = 3.975, *z* = 3.63, *p* < .001) and a 5.7‐fold greater increase than conspecifics in the mangrove (GLM, estim. = 4.655, *z* = 4.76, *p* < .001), while the increase in feeding during this period (ebb tide) did not differ between the mangrove and dock habitats (GLM, estim. = −0.755, *z* = 0.66, *p* = .507). As with tidal period, temperature impacted feeding differently between habitats. Individuals in the dock habitat increased the proportion of time they fed as temperatures rose (GLM, estim. = 0.526, *z* = 8.74, *p* < .001; Figure S2), while the opposite was observed in both the mangrove (GLM, estim. = −0.525, *z* = −8.73 *p* < .001) and salt marsh (GLM, estim. = −0.330, *z* = −2.22 *p* < .001) driving the overall negative impact of temperature on time spent feeding. Additionally, the interaction between temperature and habitat revealed that this reduction in feeding with increased temperature was greater in the mangrove than in the salt marsh (GLM, estim. = 0.195, *z* = 3.24 *p* = .002).

Movement patterns were similar to those seen in feeding as the proportion of time *A. pisonii* spent moving was not contingent upon individual size or sex (GLM, estim. = −0.021, *z* = −0.74, *p* = .458; estim. = 0.148, *z* = 1.003 *p* = .316, respectively), but was impacted by environmental factors. However, in contrast to feeding, movement decreased during ebb tide (GLM, estim. = −0.206, *z* = −4.46 *p* < .001) and increased with air temperature (GLM, estim. = 0.0433, *z* = 2.25, *p* = .024). Additionally, individuals in the mangrove spent a greater proportion of time moving (Prop. time ± *SD* = 0.116 ± 0.018) than conspecifics in the salt marsh (0.032 ± 0.037; GLM, estim. = 1.698, *z* = 9.73, *p* < .001) and dock habitats (0.040 ± 0.040; GLM, estim. = 1.322, *z* = 7.44 *p* < .001). However, movement did not differ between the salt marsh and dock habitats (GLM, estim. = −0.293, *z* = −1.73 *p* = .084). The interaction between movement and tide revealed that individuals in the dock habitat increased the proportion of time they moved after slack tide (ebb tide) as opposed to the decrease in movement in both the mangrove (GLM, estim. = −3.658, *z* = 2.49, *p* = .021) and salt marsh (GLM, estim. = −6.110, *z* = −3.44, *p* < .001) which drove the overall negative trend of reduced movement after slack tide. However, the decrease in movement during ebb tide did not differ between the mangrove and salt marsh (GLM, estim. = −2.433, *z* = −1.78, *p* = .076).

### 3.3 Exposure to thermal microhabitats

**Figure 3:**
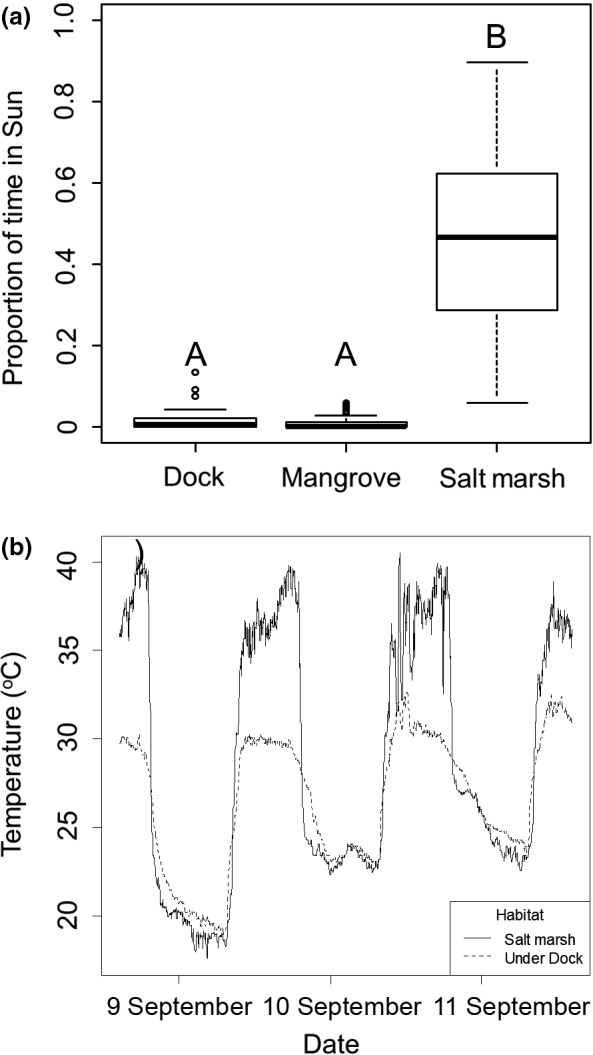
(a) Box plots comparing the proportion of time spent in sun by *Aratus pisonii* between the three habitats. Groups that are significantly different are denoted by different letters. In each box plot, and in all other box plots represented in this paper, the median is represented by a heavy line, the box represents the upper and lower quartiles, while the whiskers represent 95% of the data and circles show outliers. (b) Thermal logger data of loggers placed in the shade under a dock (dashed line) and in the open in the salt marsh (solid line) from 8 September 2016 to 11 September 2016

The thermal conditions experienced by *A. pisonii* differed greatly between habitats. Individuals observed in the dock and mangrove habitats spent a similar amount of time in the shade (Tukey's HSD, *p* = .938, Figure 3a) and more than 18‐fold less time in the sun than conspecifics in the salt marsh (ANOVA, *F*_2_ = 110.5 *p* < .001; Tukey HSD, *p* < .001, Figure 3a). This likely resulted in individuals in the mangrove and dock habitats experiencing a cooler microhabitat, as temperatures recorded during the day were as much as 10°C cooler in the shade of a dock than in the nearby salt marsh (Figure 3b). We confirmed this conclusion through the analysis of crab body temperatures obtained from the thermal photographs.

**Figure 4:**
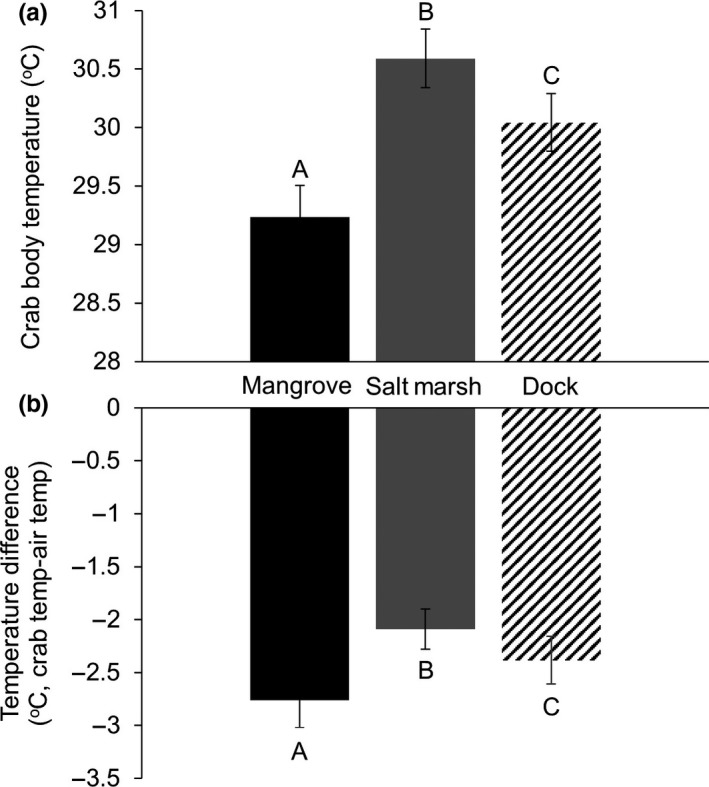
(a) Average body temperature ± *SE* of crabs in each habitat. Groups that are significantly different are denoted by different letters. (b) Differences between average crab body temperature and ambient air temperature ± *SE* in each habitat. Groups that are significantly different are denoted by different letters

Habitat played an important role in determining crab body temperature. Crabs in the salt marsh had higher body temperatures than those found in the dock and mangrove habitats (LMER, estim. = −1.1272, *t*_98_ = −2.473 *p* = .0151; estim. = −1.8366, *t*_90_ = −3.63, *p* < .001, respectively; Figure 4a). These individuals were also less able to maintain a body temperature cooler than the ambient than conspecifics in the dock and mangrove habitats (LMER, estim. = −1.2825, *t*_106_ = −3.01 *p* = .0033; estim. = −2.004, *t*_96_ = −4.21 *p* < .001, respectively; Figure 4b). Additionally, compared to conspecifics in the mangrove, crabs in the dock habitat had a higher body temperature (LMER, estim. = −0.7095, *t*_64_ = −2.427 *p* = .0181) and were less able to maintain a body temperature cooler than the ambient (LMER, estim. = −0.7180, *t*_68_ = −2.56 *p* = .0126). The temperature of crabs also increased with ambient air temperature (LMER, estim. = 0.9765, *t*_99_ = 8.99 *p* < .001) and decreased as a crab spent a greater proportion of its time in the water (LMER, estim. = −2.4725, *t*_98_ = −2.21 *p* = .0295). However, the amount of solar exposure a crab experienced did not have a significant impact on its body temperature (LMER, estim. = −0.3378, *t*_99_ = −1.64 *p* = .1036). In addition, crabs maintained body temperatures progressively cooler than ambient as the ambient temperature increased (LMER, estim. = −0.7839, *t*_105_ = −7.51 *p* < .001), as solar exposure increased (LMER, estim. = −0.4262, *t*_105_ = −2.23 *p* = .02813), and as crabs spent more time in the water (LMER, estim. = −2.6752, *t*_104_ = −2.47 *p* = .0152). Further, crabs in the salt marsh spent a greater proportion of their time in the water than conspecifics in the mangrove (ANOVA, *F*_2_ = 8.813, *p* < .001; Tukey HSD, *p* < .001; Figure S3) and dock habitats (Tukey HSD, *p* = .0087; Figure S3) which did not differ in this regard (Tukey HSD, *p* = .0732; Figure S3). This is of note as there was a positive relationship between the time a crab spent in the water and both the time it spent in the sun and its solar exposure (LM, *t*_103_ = 2.198, *p* = .030; *t*_103_ = 1.996, *p* = .048, respectively).

### 3.4 Diet and energy storage

**Figure 5:**
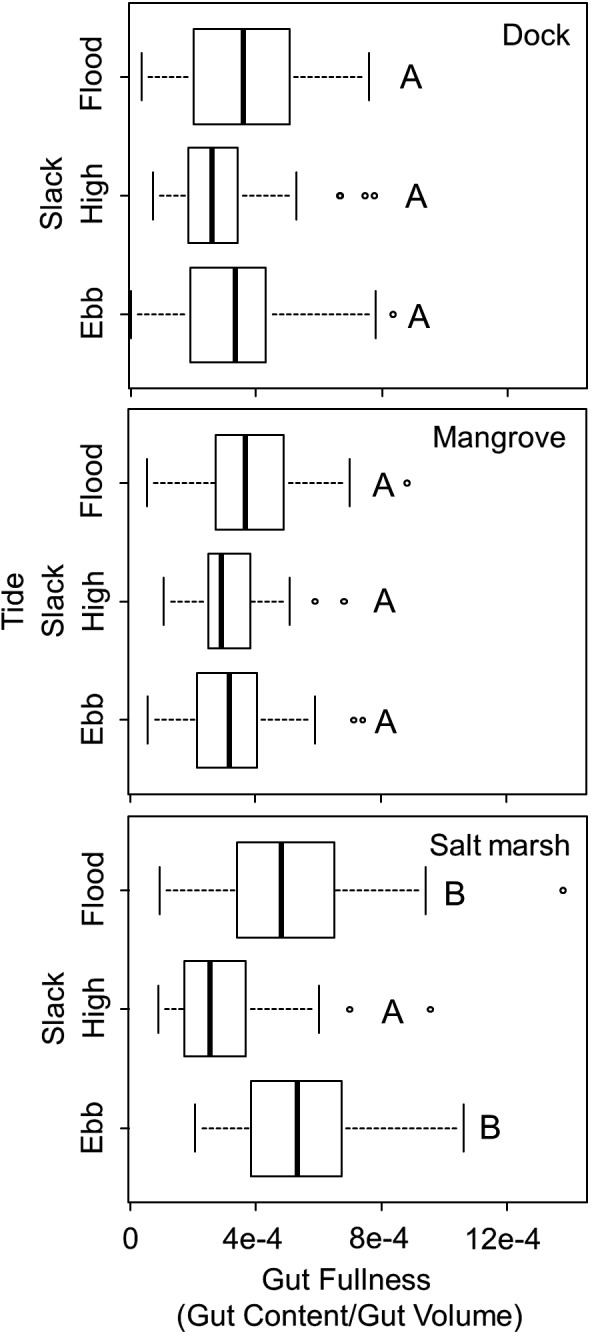
Box plots showing the gut fullness of *Aratus pisonii* by tidal period in the mangrove, salt marsh, and dock habitats. Groups that are significantly different are denoted by different letters

**Figure 6:**
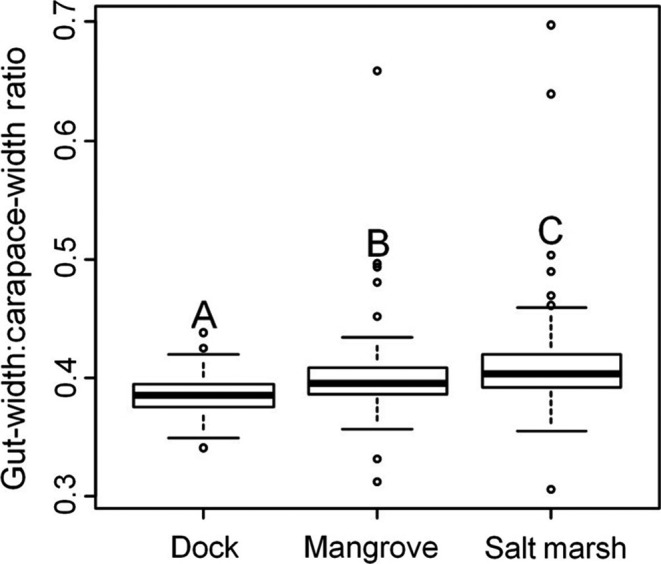
Box plots comparing the gut‐width:carapace‐width ratios of *Aratus pisonii* between the mangrove, salt marsh, and dock habitats. Groups that are significantly different are denoted by different letters. A lower gut‐width:carapace‐width ratio suggests a relatively higher proportion of animal material in the long‐term diet of the individual

The gut fullness of *A. pisonii* differed dependent on both habitat (two‐way ANOVA, *F*_2_ = 14.75, *p* < .001, Figure S4) and tidal period (two‐way ANOVA, *F*_2_ = 15.38, *p* < .001). In particular, the interaction of habitat and tidal period (two‐way ANOVA, *F*_4_ = 5.18, *p* < .001) suggests that gut fullness was dependent on a combination of these variables. When analyzed by habitat, it is clear that *A. pisonii* were able to maintain a consistent gut fullness throughout the tidal cycle in both the mangrove (Tukey HSD, *p* > .50; Figure 5) and dock habitats (Tukey HSD *p* > .50; Figure 5). However, despite an overall higher gut fullness (Tukey HSD, *p* < .001, Figure S4), crabs in the salt marsh were unable to maintain a full gut and thus were likely unable to obtain sufficient food, during the time when the rising tide restricts access to food found on the sediment or deposited by water on structure (Tukey HSD, *p* < .001; Figure 5). During other times in the tidal cycle, however, crabs in the salt marsh maintained a higher gut fullness than conspecifics in the mangrove and dock habitats (two‐way ANOVA, *F*_4_ = 5.18, *p* < .001; Tukey HSD, *p* < .01; Figure 5). In addition to unreliable foraging, *A. pisonii* in the salt marsh had a higher gut‐width:carapace‐width ratio, indicating a lower quality long‐term diet, than conspecifics in either the historic mangrove or dock habitat, where diet quality was highest (ANOVA, *F*_2_ = 20.52, *p* < .001; Tukey's HSD, *p* < .05, Figure 6).

**Figure 7:**
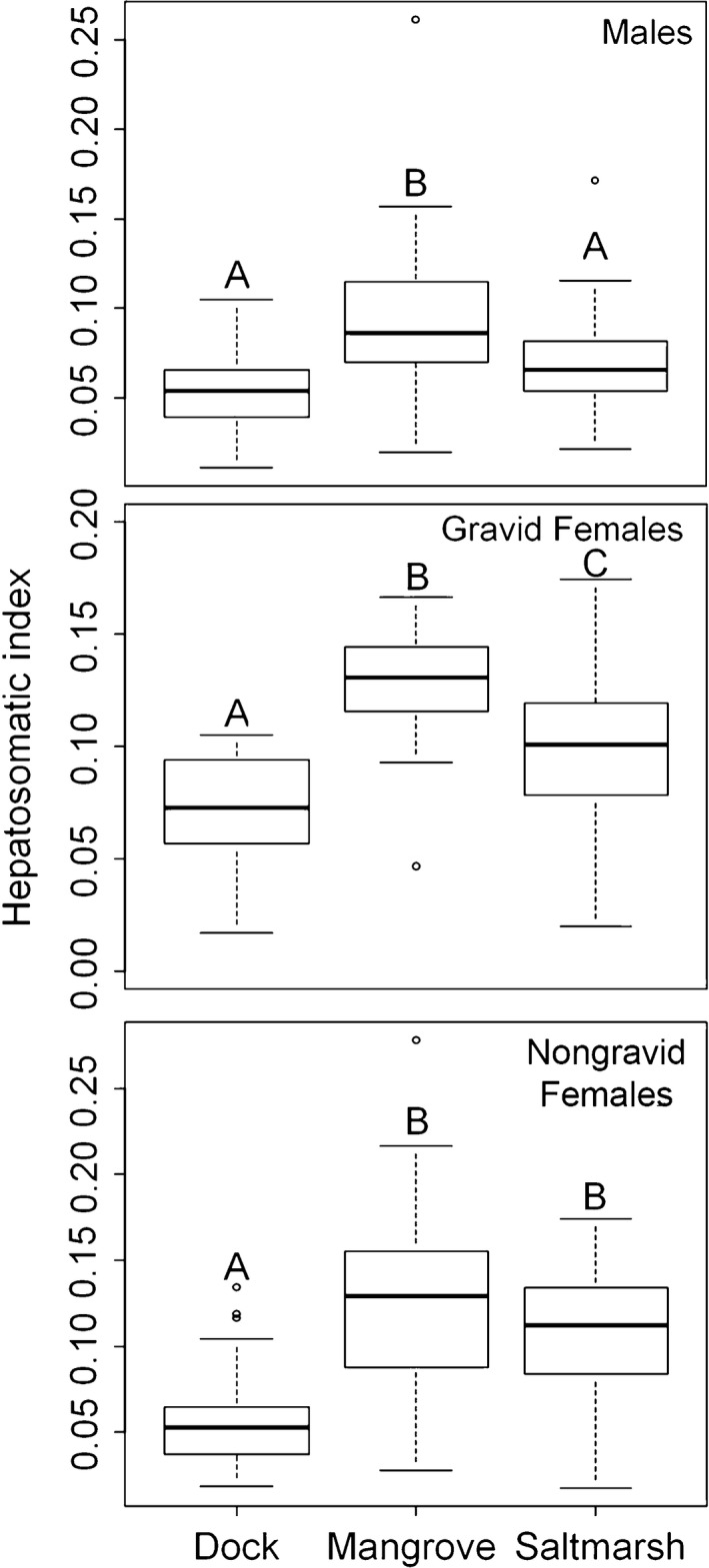
Box plots comparing the investment in long‐term energy storage, calculated as hepatosomatic index, of male, gravid female, and nongravid female *Aratus pisonii* between the three habitats. Groups that are significantly different are denoted by different letters

Proportional energetic investment into energy storage (HSI) was highest in the mangrove for both males (ANOVA, *F*_2_ = 23.27, *p* < .001; Tukey HSD, *p* < .001) and gravid females (ANOVA, *F*_2_ = 29.24, *p* < .001; Tukey HSD, *p* < .001, Figure 7). Energy storage was also greater in gravid females in the salt marsh than on docks (Tukey HSD, *p* < .001, Figure 7), but did not differ between these two habitats in males (Tukey HSD, *p* = .065, Figure 7). In nongravid females, energy storage was lowest in the dock habitat (ANOVA, *F*_2_ = 36.13, *p* < .001; Tukey HSD, *p* < .001, Figure 7) but did not differ between the mangrove and salt marsh (Tukey HSD, *p* = .060, Figure 7).

## 4 DISCUSSION

Compared to the historic mangrove, the salt marsh proved to be a suboptimal habitat for *A. pisonii* in every measured aspect of this study. Further, this study suggests that the role of the dock habitat in providing improved conditions for *A. pisonii* within the colonized salt marsh ecosystem is mixed. Yet, while docks do not provide improved conditions in every way, they do appear to provide improvements for a number of important aspects of this crab's ecology and physiology. One important benefit conferred by docks is larger body size. While there is as yet no reliable way to age these crabs (Hartnoll, 2001; Vogt, 2012), and thus no way to determine the relative impacts of age and growth rate, a larger body size is often beneficial. For *A. pisonii,* larger size confers benefits through size‐specific dominance hierarchies (Warner, 1970) and increased reproductive output (Riley & Griffen, 2017), which in turn benefits the population. Thus, greater size is an example of an individual benefit provided by an analogous habitat that may have cascading benefits for a range‐shifting species.

Understanding how analogous habitats confer general benefits, such as larger size, requires an understanding of the mechanisms that lead to those benefits. This can be explored through the examination of the precise ways in which an analogous habitat provides improved conditions. For example, the quantity and quality of an individual's diet have a direct impact on several aspects of its ecology and life history including growth (Buck et al., 2003; Griffen, Guy, & Buck, 2008), offspring quantity and quality (Green, Gardner, Hochmuth, & Linnane, 2014; Millamena & Quinitio, 2000), and bioenergetics (Charron et al., 2015; Riley, Vogel et al., 2014). Thus, an improved diet may itself be the mechanism behind other benefits including increased size. Docks clearly provide improved diet and foraging conditions to *A. pisonii* through more continuous access to a higher quality diet than elsewhere in the salt marsh. However, the high gut fullness displayed by crabs in the salt marsh when the sediment is accessible and during ebb tide suggests that they exhibit compensatory feeding through increased consumption when food is available. While compensatory feeding is common among individuals faced with poor diets, it is not always effective (Cruz‐Rivera & Hay, 2000) and may be hindered by irregular access to food in the salt marsh. In addition to regular access to food, docks provide abundant animal protein, a high‐quality food (Riley, Vogel et al., 2014), in the form of high‐density fouling communities. We regularly observed *A. pisonii* feeding on fouling organisms suggesting that animal material plays an important role in the improved diet quality of these crabs.

Similarly to diet, the thermal conditions experienced by an organism greatly impact its physiology and life history (Huey, 1991; Leffler, 1972). Thus, improved thermal conditions are a potential mechanism that could lead to other benefits including larger size (Huey, 1991; Leffler, 1972). For *A. pisonii*, docks provide a shaded thermal refuge which allows crabs to maintain a body temperature that is lower, and lower than ambient to a greater extent, than conspecifics elsewhere in the salt marsh. In fact, the extensive use of shaded areas of the dock and mangrove habitats suggests that shaded areas are preferred by *A. pisonii* and the excessive time conspecifics from the salt marsh spend in the sun is likely a result of the habitat structure, not preference. The use of thermally sheltered habitats in such areas where preferred thermal conditions are not readily available is a primary way in which species may address regional climatic shifts (Williams et al., 2008). While we focused on the ability of docks to provide crabs a cooler habitat during summer months, the ability of an analogous habitat to provide a warmer microhabitat in winter months could also be vital to a range‐shifting species.

Despite the cooler conditions provided by docks, the thermal differences observed between habitats were less than the disparity in time spent in the sun would suggest. One possibility is that crabs in the open‐structured salt marsh experience greater convective cooling due to increased wind exposure (Ortega, Mencia, & Perez‐Mellado, 2017). However, our results suggest that the lower than expected body temperature of crabs in the salt marsh is more likely a result of differences in thermoregulatory behavior. Crabs in the salt marsh appear to thermoregulate by dipping in water to cool themselves after extended time in the sun, a conclusion supported by the positive relationship between time in water and solar exposure. Indeed, a comparison of the *z*‐scored model estimates suggests that the time crabs spend in the water has the largest impact on both their body temperature and their ability to maintain a body temperature cooler than the ambient air. Additionally, dipping in water could have an additional cooling effect even after the crab emerges via evaporative cooling (Eshky, Atkinson, & Taylor, 1995), which could also be further enhanced by increased wind exposure. Indeed, in combination with the result that crabs spend more time in the water when experiencing greater solar exposure, it is possible that this could explain the unexpected negative effect of solar exposure on the difference between crab body temperature and the ambient air temperature. Thus, while exposure to the sun surely has an acute warming impact on crabs, its statistical impact is likely overpowered by the impact of cooling with water.

The change in thermoregulatory behavior in the salt marsh suggests another way in which analogous habitats may provide improved conditions in colonized ecosystems: by allowing individuals to avoid potentially costly changes in behavior. While behavioral changes often provide the first response to altered environments (Gross, Pasinelli, & Kune, 2010; Sih, Ferrari, & Harris, 2011; Wong & Candolin, 2015), they can lead to costly ecological trade‐offs. For *A. pisonii*, the need to thermoregulate may require crabs to temporarily abandon forage or shelter to move to water where they are likely exposed to higher predation (Warner, 1967; Wilson, 1989). In fact, previous work suggested that predation on large individuals may be lower in the mangrove than the salt marsh which may contribute to the size disparity between the two habitats (Riley & Griffen, 2017). It is possible that the risk of predation for large individuals is also lower on docks, particularly considering the low occurrence of small individuals (Figure S1), further contributing to the larger size of individuals found there. However, while docks may allow *A. pisonii* to avoid risky thermoregulatory behavior, crabs found there exhibit foraging behavior that differs from crabs in the mangrove and is similar to conspecifics elsewhere in the salt marsh. Crabs in the dock and salt marsh habitats increase their feeding as the tide falls suggesting they feed heavily on food that is either deposited on structure or submerged at high tide. This differs from conspecifics in the historic mangrove which feed on continuously accessible mangrove leaves. Like dipping in water to thermoregulate, following receding water to feed may increase the risk of predation by aquatic predators (Warner, 1967; Wilson, 1989). Thus, the ability of docks to allow *A. pisonii* to avoid potentially dangerous behavioral changes is mixed.

Foraging behavior is not the only way docks fail to provide improved conditions for *A. pisonii*. In particular, the proportion of energy stored by crabs in the three habitats differed in unexpected ways. While the investment into energy storage (HSI) was lower in the salt marsh than the historic mangrove habitat, it was lower still in crabs found on docks. This is particularly perplexing when considering the larger size and improved diet of crabs on docks. It is possible that the differences in diet observed between habitats play a role in the ability of *A. pisonii* to convert consumed energy into stored energy. Alternatively, some unknown energetic expense or trade‐off in the dock habitat may lead to a decrease in energy storage. In any event, the energy storage of *A. pisonii* warrants further study and suggests that crabs on the docks likely have different patterns of energy use than those in the surrounding salt marsh ecosystem. Given the metabolic costs for crabs of storing lipids in the hepatopancreas (Griffen, 2017), the lower HSI seen in crabs on the docks could be beneficial for individuals and may reflect improved energetic efficiency for crabs using this habitat type.

While docks appear to provide several important benefits to *A. pisonii* in the colonized salt marsh ecosystem, their role as an analog to the mangrove is clearly mixed. Yet, what docks do represent is a relatively understudied aspect of range shift ecology: the role of anthropogenic habitat analogs in providing improved conditions within suboptimal colonized natural ecosystems. However, a number of studies have proposed implementing artificial habitats, or habitat modification, to minimize the exposure of vulnerable species to stressful changing conditions in their historic ecosystems (Shoo et al., 2011; Williams et al., 2008). Such proposals have included installing microhabitat refuges and sprinklers for amphibians (Shoo et al., 2011), artificial breeding structures (Shoo et al., 2011), shade cloths (Mitchell et al., 2008), and general habitat restoration using artificial structures such as burrows (Souter et al., 2004) and formed concrete (Webb & Shine, 2000). However, the use of anthropogenic habitats in natural ecosystems that a species has never before inhabited has garnered little discussion.

The construction of artificial habitats in unsuitable ecosystems to help/encourage range shifts has received some discussion as a facet of adaptive management strategies (Hoegh‐Guldberg et al., 2008). Additionally, there has been a robust discussion of the use of corridors to aid species in their climate‐induced range shifts (Hannah, 2001; Krosby et al., 2010). In fact, increasing ecological connectivity through cities and other unfavorable habitats to encourage the movement of species between natural areas has been identified as critical to the ability of many species to persist in the face of changing climatic conditions (Krosby et al., 2010; Williams, Eastman et al., 2014; Williams, Lundholm et al., 2014). Such discussions tend to focus on creating or preserving natural corridors between natural areas (Hannah, 2001; Krosby et al., 2010). In contrast, anthropogenic habitat analogs may increase, rather than impede, the success and rate of range shifts. While there has been some exploration of green roofs (Williams, Eastman et al., 2014; Williams, Lundholm et al., 2014 and references therein), gardens (Goddard, Dougill, & Benton, 2010), street‐side vegetation (Swan, Pickett, Szlavecz, Warren, & Willey, 2011), and other anthropogenic “stepping‐stone” refuges (Chester & Robson, 2013; Gledhill, James, & Davies, 2008; Santoul, Gaujard, Angélibert, Mastrorillo, & Céréghino, 2009) in facilitating movement through cities and other unfavorable habitat, this work has largely focused on biodiversity conservation and movement between habitable areas as opposed to range shifts (but see Grant, 2006). Yet, anthropogenic structures which were not specifically designed as habitat could increase the permeability of the habitat matrix during range shifts by providing more favorable habitat than the surrounding ecosystem. Even if anthropogenic habitat analogs do not increase the rate of a range shift, their ability to provide improved conditions could prove vital to the success of range‐shifting species in colonized ecosystems.

As climate change continues to force or encourage species to colonize new ecosystems, it will be increasingly important to understand how these shifting species are impacted by habitats with which they have no ecological or evolutionary experience. The role of anthropogenic habitats as habitat analogs may play a crucial role in the outcome of range shifts. Thus, the existence of anthropogenic habitat analogs should be included in analyses of the vulnerability of species to climate change (see Williams et al., 2008 for a framework for such an analysis). Ultimately, the individual benefits conferred by docks suggest that they likely have a positive impact on the population of *A. pisonii* in the salt marsh. Therefore, this study suggests that anthropogenic habitats have the potential to play an important role in providing improved conditions to range‐shifting species experiencing suboptimal conditions in colonized ecosystems. While no habitat analog is likely to ameliorate all negative novel interactions experienced by range‐shifting species, amelioration of even a small number of negative impacts will likely be beneficial to both individuals and populations. If the patterns that we document are general across systems, then anthropogenic habitats may play an important facilitative role in the range shifts of species with continued climate change.

## AUTHOR CONTRIBUTIONS

ZJC and BDG conceived and designed the experiments. ZJC conducted field work and behavioral observations. ZJC and SRD collaborated on dissections and thermal image analyses. ZJC performed statistical analyses. ZJC wrote the manuscript. BDG provided editorial advice and guidance throughout the project.

## CONFLICT OF INTEREST

None declared.
