# Supplementary figures and images for: An anthropogenic habitat within a suboptimal colonized ecosystem provides improved conditions for a range‐shifting species
Source: Ecol Evol. 2018 Jan 1;8(3):1521–33. doi: 10.1002/ece3.3739 (PMC5792588; doi:10.1002/ece3.3739)

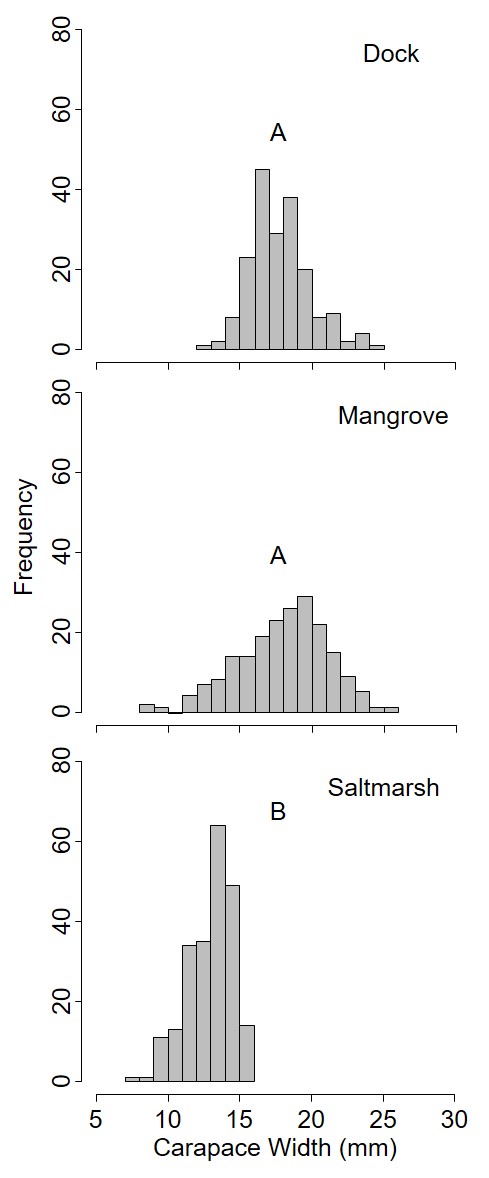

Supplement: Supplementary file 1 [file ECE3-8-1521-s001.jpg]

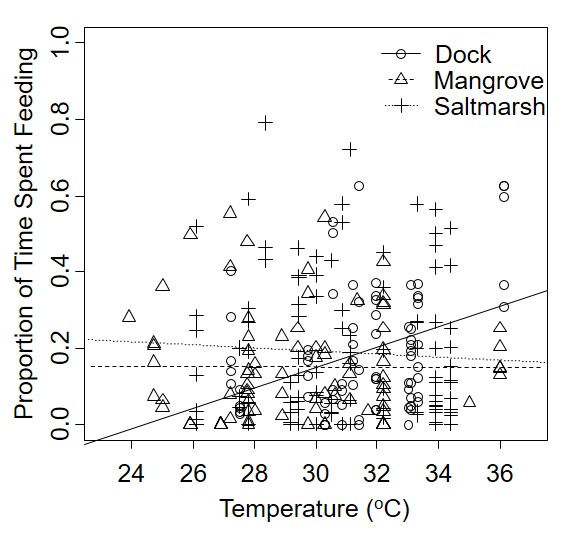

Supplement: Supplementary file 2 [file ECE3-8-1521-s002.jpg]

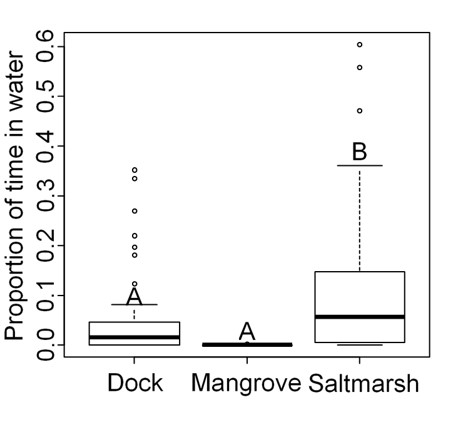

Supplement: Supplementary file 3 [file ECE3-8-1521-s003.jpg]

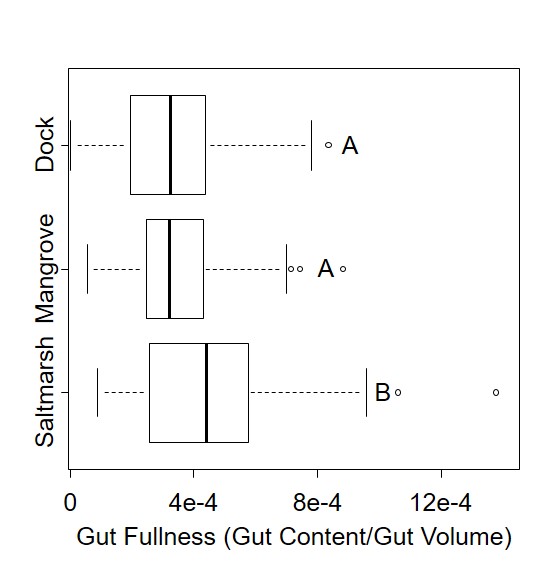

Supplement: Supplementary file 4 [file ECE3-8-1521-s004.jpg]
