# Supplementary material for: An anthropogenic habitat within a suboptimal colonized ecosystem provides improved conditions for a range‐shifting species
Source: Ecol Evol. 2018 Jan 1;8(3):1521–33. doi: 10.1002/ece3.3739 (PMC5792588; doi:10.1002/ece3.3739)
Supplement: Supplementary file 5 [file ECE3-8-1521-s005.docx]

**Appendix 1**

**Appendix Tables**

**Table A1** Name, habitat type, and location of site in latitude-longitude coordinates. The groups of crabs observed for behavior at each site is also included

| Site | Habitat | Lat-Long | Groups of Crabs Observed |
| --- | --- | --- | --- |
| Round Island Park | Mangrove | 27^o^33’33”N  80^o^19’53”W | 1 |
| Pepper Park | Mangrove | 27^o^29’42’N  80^o^18’12”W | 3 |
| Oslo Park | Mangrove | 27^o^35’14”N  80^o^21’55”W | 2 |
| North Causeway Park | Mangrove | 27^o^28’28”N  80^o^19’12”W | 2 |
| Bear Point | Mangrove | 27^o^25’48”N  80^o^17’10”W | 2 |
| GTM NERR | Saltmarsh | 30^o^0’49”N  81^o^20’42”W | 12 |
| Anastasia State Park | Saltmarsh | 29^o^52’40”N  81^o^16’32”W | 4 |
| Palm Valley | Dock | 30^o^7’57”N  81^o^23’8”W | 5 |
| Yacht Club | Dock | 29^o^53’9”N  81^o^17’8”W | 3 |

**Appendix Figure Legends**

**Fig A1** Size frequency distributions of *A. pisonii* in each of the three habitat types. Groups that are significantly different are denoted by different letters

**Fig A2** Relationship of the proportion of time crabs in each habitat spent feeding and ambient temperature. Lines show slopes of relationships for each individual habitat

**Fig A3** Boxplots of the proportion of time spent in the water by crabs in each of the habitats. Groups that are significantly different are denoted by different letters

**Fig. A4** Boxplots comparing the tide-independent gut fullness of *A. pisonii* between habitats. Groups that are significantly different are denoted by different letters
